# Structural Analysis of the Pin1-CPEB1 interaction and its potential role in CPEB1 degradation

**DOI:** 10.1038/srep14990

**Published:** 2015-10-12

**Authors:** Constanze Schelhorn, Pau Martín-Malpartida, David Suñol, Maria J. Macias

**Affiliations:** 1 Institute for Research in Biomedicine (IRB Barcelona), The Barcelona Institute of Science and Technology (BIST), Baldiri Reixac 10, Barcelona, 08028, Spain; 2 Catalan Institution for Research and Advanced Studies (ICREA), Passeig Lluís Companys 23, Barcelona, 08010, Spain

## Abstract

The Cytoplasmic Polyadenylation Element Binding proteins are RNA binding proteins involved in the translational regulation of mRNA. During cell cycle progression, CPEB1 is labeled for degradation by phosphorylation-dependent ubiquitination by the SCF^β−TrCP^ ligase. The peptidyl-prolyl isomerase Pin1 plays a key role in CPEB1 degradation. Conditioned by the cell cycle stage, CPEB1 and Pin1 interactions occur in a phosphorylation-independent or -dependent manner. CPEB1 contains six potential phosphorylatable Pin1 binding sites. Using a set of biophysical techniques, we discovered that the pS210 site is unique, since it displays binding activity not only to the WW domain but also to the prolyl-isomerase domain of Pin1. The NMR structure of the Pin1 WW-CPEB1 pS210 (PDB ID: 2n1o) reveals that the pSerPro motif is bound in *trans* configuration through contacts with amino acids located in the first turn of the WW domain and the conserved tryptophan in the β3-strand. NMR relaxation analyses of Pin1 suggest that inter-domain flexibility is conferred by the modulation of the interaction with peptides containing the pS210 site, which is essential for degradation.

The Cytoplasmic Polyadenylation Element Binding (CPEB) proteins activate RNA translation, promoting the elongation of the poly(A) tail of messenger RNAs. CPEB proteins can also act as repressors, stabilizing the closed-loop structure of the mRNAs and thereby inhibiting translation^1^,^2^. The transition from translational inhibition to promotion of the elongation of the poly(A) tail is a complex process in which the phosphorylation of the CPEB proteins plays a crucial role^3^. Phosphorylation is also a prerequisite to the labeling of these proteins for controlled degradation^3^,^4^,^5^. During oocyte maturation, partial destruction of CPEB1 is necessary for the transition from Metaphase I (MI) to Metaphase II (MII) and for the functional substitution of CPEB1 by CPEB4^6^. This mechanism requires the phosphorylation of CPEB1 to create the binding site for the SCF^β-TrCP^ ubiquitin ligase and guarantees the polyadenylation of various CPE-containing mRNAs, a process regulated by the levels of different CPEBs *in vivo* in a time-specific manner.

The CPEB protein family consists of four members. All CPEBs are characterized by the presence of a pair of highly conserved RNA binding domains (RRM domains) and a ZZ-domain at the C-terminus (Fig. 1a). CPEBs also contain a protein-protein interaction site located at the N-terminus, which is highly divergent among the different members of the family. The RNA binding characteristics of CPEB1 and CPEB4 have been recently characterized to atomic detail^7^,^8^. The structural information has revealed that efficient recognition of the CPE sites is achieved using the pair of RRM domains working as a team, increasing the specificity by augmenting the protein-binding surface. However, the information available on the protein-protein binding site of CPBEs is more limited. CPEB1 degradation during maturation requires a Cdc2-mediated phosphorylation of six Serine residues (138, 144, 184, 210, 248, and 423) in Ser-Pro motifs, with the phosphorylation of Ser210 being essential for subsequent CPEB1 degradation^9^. *In vivo*, the processes of phosphorylation and ubiquitination occur very rapidly^10^ with the *trans* configuration of the pSer-Pro motifs being crucial for SCF^β-TrCP^ ubiquitin ligase recognition^11^,^12^,^13^. In this context, it is not surprising that phosphorylated CPEB1 has recently been identified as a target for the peptidyl-prolyl isomerase Pin1 and that the isomerase protein regulates CPEB1 destruction^14^. It has also been observed that the interaction of CPEB1 and Pin1 can occur even in the absence of detectable CPEB phosphorylation^14^ suggesting that the pre-association of Pin1 with its unphosphorylated target protein might be essential for ensuring the presence of Pin1 to interact with the phosphorylated substrate when it is required.

Prompted by these observations, we sought to characterize the interaction of Pin1 with N-terminal fragments of CPEB1, phosphorylated and unphosphorylated, at an atomic detail. For this purpose, we prepared recombinant proteins and phosphorylated peptides and applied a combination of high-resolution NMR spectroscopy and complementary biophysical techniques, including Ion-Mobility coupled to Mass Spectrometry.

It is well established that Pin1-target recognition is achieved by the interaction of its WW domain with motifs present in the target proteins. Therefore, we first examined whether the WW domain interacts with recombinant fragments of CPEB1, in the basal (unphosphorylated) state. We used NMR to study the interaction of both the WW domain and the full-length Pin1 with synthesized peptides containing the potential six phosphorylatable Pin1 binding sites in CPEB1. In addition, we characterized the effects of peptide binding on the inter-domain flexibility of Pin1, by measuring the NMR relaxation properties of the full-length Pin1 in complex with CPEB1 pS210 peptide, and determined the solution structure of the Pin1 WW domain in complex with the CPEB1 pS210 motif (PDB ID: 2n1o). Our results, together with the available information in the literature, contribute to a better understanding of the role of Pin1 in ubiquitination and CPEB1 protein degradation.

## Results

### Pin1 WW domain associates with CPEB1 prior to its phosphorylation

**Figure 1:**
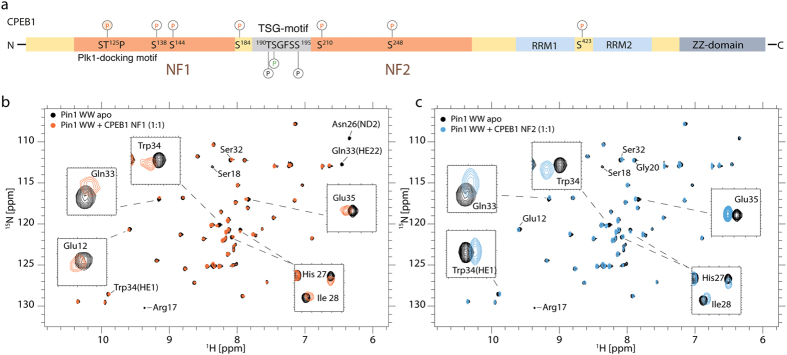
Phosphorylation independent interaction of CPEB1 and Pin1 WW. (**a**) Schematic representation of the topology of CPEB1 including the regulatory phosphorylation sites. Superimposition of ^1^H, ^15^N - HSQC spectra of Pin1 WW domain (black) and in complex with (**b**) CPEB1 NF1s (orange) and (**c**) CPEB1 NF2 ∼1 molar equivalents.

CPEB1 proteins display high sequence conservation in vertebrates (Supplementary Fig.1). To gain insight into the association of Pin1 with CPEB1 in the basal state, we prepared two recombinant fragments named NF1 (88–183) and NF2 (196–293), selected from the N-terminal region of CPEB1 (Fig. 1a). The boundaries of these constructs were chosen to be similar to that used by *Nechama et al.*^14^, and were extended to include Ser210, known to be essential for degradation after phosphorylation by Cdc2^9^. Since target recognition of Pin1 is achieved by the interaction of the WW domain with the protein partners, we prepared an ^15^N-labeled WW domain sample and monitored the chemical shift perturbations induced in the WW domain by the presence of the ligands (1:1 WW: ligand ratio) using HSQC experiments (Fig. 1b,c). These experiments reveal that, despite the low affinity associations, residues of the WW domain are clearly affected by the presence of the CPEB 1 fragments. Attempts to saturate the NMR titration using high ligand excess were precluded due to the aggregation tendency of the NF1 and NF2 fragments. To obtain affinity values for these interactions, we applied ITC titrations. We performed these titrations (data not shown) using the Pin1 WW domain as the titrant and either the NF1 or the NF2 fragments as titrates since the Pin1 WW domain is more soluble at higher concentrations than the NF1 or NF2 fragments. Our results indicate that the binding affinities lie in the high μM up to the mM range, since the titrations could not be efficiently saturated. The HSQC experiments revealed that similar residues in the Pin1 WW domain showed chemical shift perturbations due to the presence of either NF1 or NF2 ligands. In both complexes, the affected residues of the WW domain are located at the beginning of the first β-strand (Glu12; we use one-letter code for CPEB1 amino acids and three-letter code for Pin1 residues) and second loop (His27, Ile28), which are in spatial proximity to each other, and also residues at the end of the third β-strand (Ser32, Gln33, Trp34 and Glu35). It its noteworthy that the amide resonances from Arg17 and Ser18 disappear when CPEB1 associates with the Pin1 WW domain. In addition to the common changes induced by both ligands, we observed that the side-chain resonances of Asn26 and Gln33 are undetectable in the spectrum of the complex with NF1. We attribute the line broadening to these residues being in proximity with the ligand, with binding occurring in the intermediate exchange regime on the NMR time scale. Overall, the data obtained by NMR and ITC corroborate the interactions revealed in experiments using mammalian cells^14^ and indicate that the WW domain of Pin1 and the unphosphorylated CPEB1 N-terminus interact with weak affinity (Fig. 1b,c).

### Phosphorylation enhances the affinity of the Pin1-CPEB1 interaction

**Figure 2:**
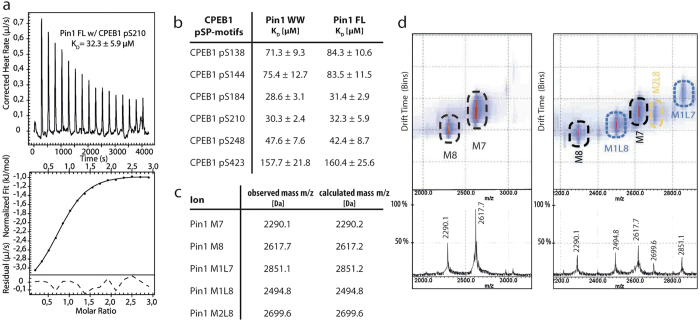
CPEB1 pS210 and pS184 are the preferred Pin1 binding sites. (**a**) ITC curves of Pin1 full-length with CPEB1 pS210 and (**b**) affinity values obtained for the titrations with Pin1 WW and Pin1 full-length. (**c**) Exact ion-masses (m/z) of both observed and theoretical values in Ion-Mobility-Mass Spectrometry experiments. (**d**) A region of the IM-MS data for Pin1 full-length in its apo form (left) and in complex with CPEB1 pS210 (right). Each ion was assigned to a given species based on its characteristic mobility and mass-to-charge ratio. Abbreviations used are M (Monomer, apo state), M1L (Monomer with one ligand) and M2L (Monomer with two ligands). Numbers following the species’ name indicate the ionization state. In the free state solely monomers are detected. In complex two types of species are detectable: monomeric Pin1 bound to one ligand (ions M1L7 and M1L8) and in complex with two ligands (ion M2L8).

Pin1 interacts with phosphorylated CPEB1 in *Xenopus* oocytes, as well as in mammalian cells^14^. In addition, several studies have indicated that the inter-domain communication between the WW and the catalytic domain in Pin1 is important for the activity *in vivo*^15^. Therefore, to mimic the *in vivo* scenario, we measured the affinities of the interactions between recombinant proteins (the isolated WW domain and also the full-length protein) with several peptides containing the phosphorylation sites within CPEB1. These peptides were synthesized using Fmoc Solid-Phase Peptide Synthesis (SPPS) (Supplementary Fig 2). ITC titrations show that all the CPEB1 peptides prepared interacted with Pin1 recombinant proteins *in vitro*. All affinities were found in the low μM range (Fig. 2a,b), with the exception of the pS423 site of CPEB1, located in the linker region between the RRM domains, whose affinity was considerably lower than the rest. Our results reveal that the peptides containing pS184 and pS210—the latter known to be essential for CPEB degradation—display the best affinities for Pin1 *in vitro*. The affinity of the phosphorylation-dependent interaction was about 10-fold higher than that of the unphosphorylated CPEB N-terminal counterparts. In addition, in most of the peptides examined, similar binding affinities and stoichiometries (1:1) were observed using either the full-length Pin1 protein or the truncated WW domain. Only when analyzing the interaction of CPEB1 pS210 and the full-length Pin1 protein we obtained a stoichiometry below 1 (n = 0.85), which cannot be attributed to systematic errors in the determination of the protein concentration. This discrepancy might indicate that in addition to the main complex consisting of a 1:1 protein/peptide ratio, a small population with a 1:2 stoichiometry is also present in solution.

In order to characterize the presence of complexes of different stoichiometries, we applied Ion Mobility-Mass Spectrometry (IM-MS) since this technique is valuable to detect the populations of different protein complexes at concentrations used in ITC experiments^7^,^16^. The analysis of the IM-MS data revealed the presence of monomeric species in the apo Pin1 form (Fig. 2c, left panel, and Fig. 2c) or complexes with one ligand (M1L) or with two ligands (M2L) (Fig. 2c, right panel). All together, these results indicate that the affinities and stoichiometries obtained by ITC reflect the contribution from two species: the 1:1 complex that corresponds to one ligand bound to the protein, and the 1:2 complex where a second one is bound.

### Identifying the binding site for CPEB1 phosphopeptides within Pin1

**Figure 3:**
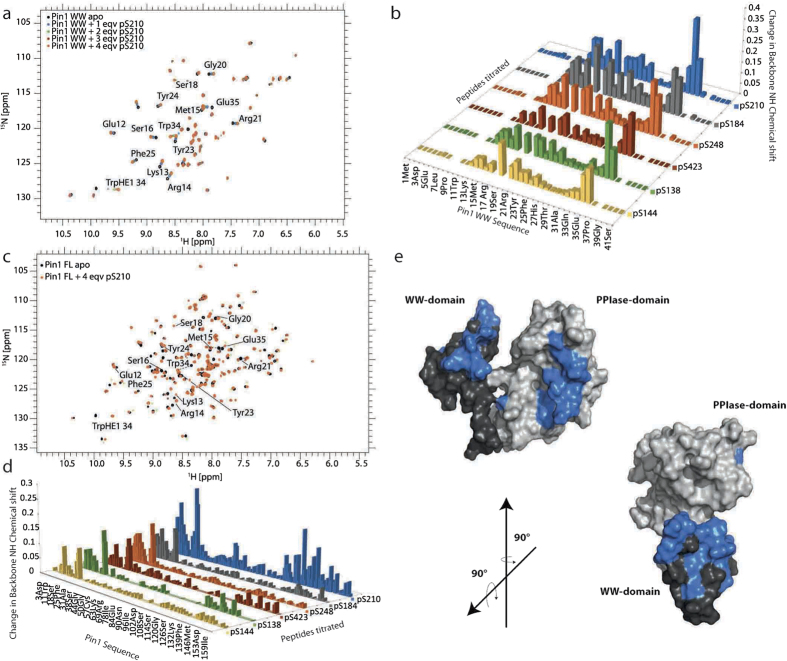
Characterization of the phosphorylation dependent CPEB1-Pin1 interaction by NMR. (**a**) Superimposition of ^1^H, ^15^N - HSQC spectra of Pin1 WW domain (black) and in complex with CPEB1 pS210. Titration steps with 1–5 molar equivalents are shown in blue, green, red and orange, respectively. The most affected residues upon binding are labeled. (**b**) Chemical shift perturbations of Pin1 WW domain upon binding to CPEB1 pS210 (blue), pS184 (grey), pS248 (orange), pS423 (red), pS138 (green) and pS144 (yellow). The CSPs are plotted versus residue numbers. (**c**) Superimposition of ^1^H, ^15^N - HSQC spectra of Pin1 full-length in its apo state (black) and in complex with CPEB1 pS210 (4 equivalents, orange). Affected residues from the catalytic domain are marked with an asterisk. (**d**) Chemical shift perturbations of Pin1 full-length upon binding to CPEB1 pS210 (blue), pS184 (grey), pS248 (orange), pS423 (red), pS138 (green) and pS144 (yellow). The CSPs are plotted versus residue numbers. Within the catalytic domain significant chemical shift perturbations are only observed for the complex with CPEB1 pS210 peptide. (**e**) NH chemical shift perturbations (coloured in blue) are mapped on the solution structure of Pin1 (PDB entry: 1NMV) shown in surface representation. The WW-domain is displayed in dark grey, PPIase domain in light grey.

To further study, these interactions, we monitored the 1H- ^15^N amide chemical shift perturbations in full-length Pin1 upon titration of the phosphorylated CPEB1 peptides and compared them to the shifts induced in the isolated WW domain. Based on the backbone assignments of Pin1 WW previously obtained in our laboratory (BMRB entry: 17545) and the assignment of Pin1 full-length published (BMRB entry: 5305), we identified all amide resonances. In all titrations, the binding kinetics observed were in the fast to intermediate range on the NMR chemical shift time scale, since most of the chemical shifts changed continuously upon titration, with only a few signals disappearing and reappearing upon saturation of the interaction (Fig. 3a, for example Trp34 Hε_1_).

In general, chemical shift perturbations observed within the WW domain displayed a similar pattern for all the ligands tested. Furthermore, chemical shift perturbations in residues corresponding to the WW domain are similar upon titration with the peptides independently of using the full-length Pin1 protein or to the isolated WW domain (Fig. 3b,d). We displayed the chemical shift changes on the NMR structure of full-length Pin1 (PDB code: 1 nmv) (Fig. 3d). Affected residues in the WW domain were located mainly at the end of the β1 -strand (Ser16), the loop region between β1- and β2 -strand (Ser18, Gly20), the β2 -strand (Arg21, Tyr23, Phe25), and the residues at the end of the β3-strand (Trp34, Glu35). Interestingly, CPEB1 pS210 was the only ligand causing significant perturbations also within the catalytic domain. We observed perturbations in the PPIase active site (Leu122, Gly123, Phe134) and the proline substrate-binding pocket (Gly128), in the domain interface, mainly on β6 and α4 and in the catalytic loop (Ser72, Ser73, Trp74). These results corroborate the ones obtained by ITC and Ion Mobility-Mass Spectrometry. Taken together the experiments indicate that the 1:2 complex detected by IM-MS consists of one ligand bound to the WW domain and the second recognized by the catalytic domain.

### Dynamics of Pin1 upon binding of CPEB1 pS210

**Figure 4:**
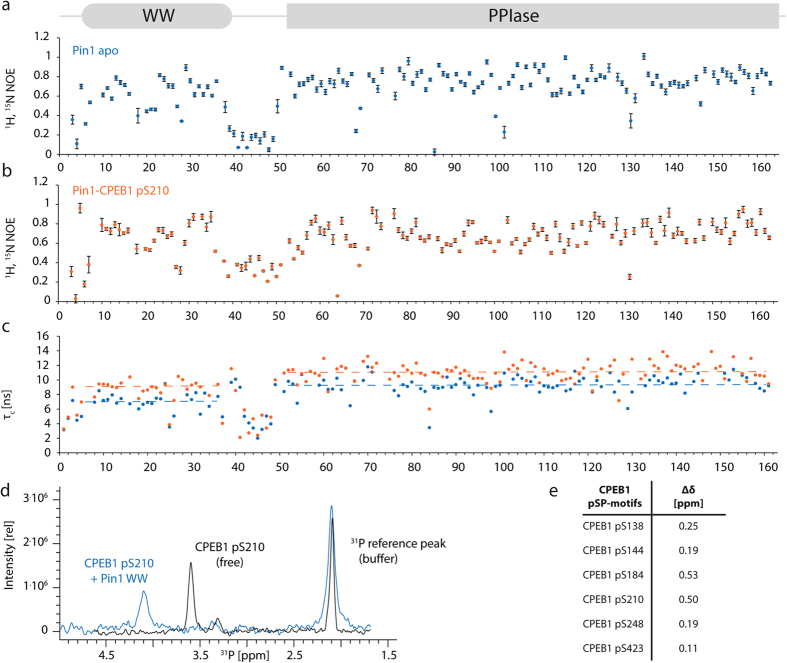
Dynamics of the Pin1-CPEB1 pS210 complex and the direct involvement of the phosphate group in binding. (**a**) Measurement of ^1^H, ^15^N NOE values for the apo Pin1 and (**b**) in complex with the pS210 ligand. (**c**) Local rotational correlation times τ_c_ were calculated from the T_1_/T_2_ ratio. Homogeneous values along the single domains were obtained: T_1_^WW, apo^ = (612.7 ± 53.6) ms, T_1_^Cat,apo^ = (792.7 ± 81.8) ms, T_2_^WW,apo^ = (113.5 ± 6.9) ms and T_2_^Cat,apo^ = (85.4 ± 13.1) ms; T_1_^WW,pS210^ = (767.1 ± 80.1) ms, T_1_^Cat,,pS210^ = (913.5 ± 91.3) ms, T_2_^WW,pS210^ = (94.1 ± 5.8) ms and T_2_^Cat,pS210^ = (74.8 ± 6.7) ms. (**d**) 1D- ^31^P NMR titration experiment of CPEB1pS210 with Pin1 WW domain. The black spectrum shows the free peptide; in blue the saturated complex with Pin1 WW is displayed. The phosphate buffer reference peaks hardly shows any chemical shift perturbation. (**e**) Chemical Shift Perturbations of the phosphate group of several CPEB1 peptides upon titration with Pin1 WW domain.

Among the six phosphorylated peptides examined, CPEB1 pS210 was the only peptide to induce significant chemical shift perturbations in the catalytic domain of Pin1. To characterize the dynamic properties of this complex, we analyzed the relaxation properties of Pin1 in the unbound state and in complex with the pS210 peptide. ^1^H-^15^N NOE, T_1_ and T_2_ (longitudinal and transverse) relaxation times showed that, in both samples, the catalytic domain and the WW domain were well structured (Fig. 4a–c). Within the catalytic domain, the loop comprising residues Gln66-Lys77 recognizes the phosphate moiety of the substrate. This area, in particular Ser67-Arg69, is a rather flexible loop within the domain, which upon binding to CPEB1 pS210, becomes slightly more rigid. However, the flexible nature of this catalytic loop region remained in the presence of the substrate. The linker connecting the WW domain and the catalytic site of Pin1 showed high flexibility in the apo and bound state (Fig. 4a,b). Although the ^1^H-^15^N NOE values obtained in the complex were higher than those found in the unbound state, they nevertheless remained in the range of values indicating a highly flexible region. This observation suggests that the linker region is not involved in interactions neither with the domains nor with the peptide. Using NMR relaxation experiments and the concept of inter-domain interaction parameter^17^, we measured the extent to which the flexibility adjusts upon ligand binding. The interaction parameter can adopt values between 0 (for fully independent domains) and 1 (when both domains are tumbling as a single unit) (Supplemental information). Using a theoretical τ_c_^FL-rigid^ of 11.6 ns and the experimentally determined values of τ_c_^WW^ = 7.3 ns and τ_c_^Cat^ = 9.4 ns, the inter-domain interaction parameter was found to be 0.46 for Pin1 in the unbound state. The τ_c_ values were larger than expected for two fully independent tumbling domains of their respective molecular weights, further supporting the notion of an inter-domain interaction (Fig. 4c). Upon binding of CPEB1 pS210, the correlation time for the WW domain increased to τ_c_^WW^ = 9.2 ns, and to τ_c_^Cat^ = 10.9 ns for the catalytic domain. The higher value of the inter-domain interaction parameter (*x*_*complex*_ = 0.7) indicates an increased restriction of the flexibility of the two domains upon binding of the ligand. However, the complex does not tumble as a single unit and residual flexibility remains. Similar observations have been made using Pintide, a peptide designed to be an optimal substrate for Pin1^17^.

### The phosphate group is directly involved in binding: Structure of the Pin1 WW-CPEB1 pS210 complex

**Figure 5:**
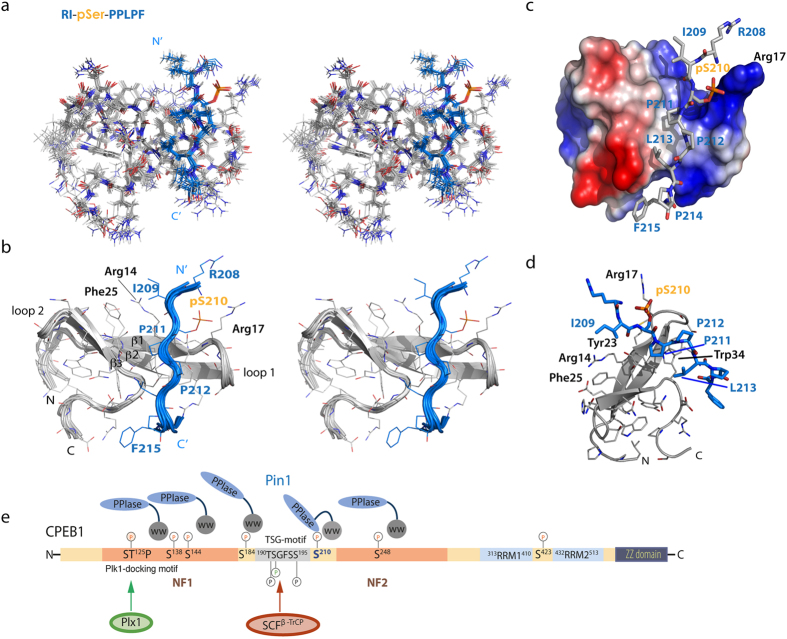
Pin1 WW—CPEB1 pS210 NMR solution structure and a model for the CPEB1 degradation scenario. (**a**) Stereo view of the best-fit backbone superposition of the ten lowest energy structures after water refinement in sticks representation and (**b**) cartoon representation. Pin1 WW is displayed in grey; the ligand CPEB1 pS210 is colored in blue. (**c**) Lowest-energy structure of the Pin1 WW - CPEB1 pS210 complex in surface charge distribution. (**d**) Cartoon representation of the Pin1 WW domain. The domain is colored in grey and residues are labeled in the 3-letter code, the peptide is colored in blue with its residues labeled in the 1 letter code. (**e**) A model for the Pin1-phospho CPEB1 interaction including the molecular mechanisms of CPEB1 degradation.

In order to illustrate the effect of the phosphorylation of CPEB1 peptides on the interaction with the full-length Pin1 or with the isolated WW domain, we performed sets of 1D-^31^P NMR-based titrations, where changes in the ^31^P chemical shift of the phosphate group were monitored (Fig. 4d). The largest chemical shift perturbations were observed for the pS210 and pS184 CPEB1 peptides (Fig. 4e), thereby supporting a direct role of the phosphate group in the interaction. To characterize the interactions of the WW domain and the phosphorylated peptides of CPEB1 in detail, we focused on the pS210 complex with the WW domain. The structure was calculated on the basis of 539 NMR-derived experimental constraints, excluding intra-residual NOEs. The 20-lowest energy ensemble exhibited atomic r.m.s. deviations of 0.93 ± 0.02 and 1.38 ± 0.2 Å with respect to the mean coordinate positions for the backbone (N, C_α_, C′) and all heavy atoms, respectively (Table 1). A stereoview of the ensemble of conformers in sticks and in ribbon representation are depicted in Fig. 5a,c, respectively. The solution structure of the complex (PDB ID: 2n1o) revealed that the Pin1 WW domain adopts a canonical three-stranded anti-parallel β-sheet fold^16^ with the CPEB1 pS210 peptide oriented from the N- to the C-terminus (Fig. 5). In the complex with CPEB1 pS210, abundant inter-molecular NOEs were observed, including contacts of P212 and L213 with Trp34 of Pin1. We use one-letter code for CPEB1 amino acids and three-letter code for Pin1 residues. The side chain of Arg17, located in the β1-strand, participates in the coordination of the phosphate group of pS210-CPEB1. Furthermore, P211 is bound in *trans* configuration between the aromatic side-chains of Tyr23 and Trp34 lying on the β2- and β3-strand, respectively. Additionally, several NOEs between P212 and L213 with Trp34 and of the I209 side-chain with the Tyr23 and Phe25 aromatic rings were also detected. As observed in Pin1 WW complexes with other p(S/T)P-motifs, Arg17 plays a key role in phosphate binding^16^,^18^,^19^. However, in the complex of Pin1 WW1 domain and the pS210 peptide, the phosphate group is accommodated in the region of the turn connecting the β1–β2 strands (Fig. 5c) and, unlike in other complexes^16^,^18^, no contacts between Arg14 (located at the beginning of the first strand, Fig. 5b,d) and the peptide were detected. The binding of Pin1 and CPEB1 resembles the structure of the Nedd4L WW3 in complex with the di-phosphorylated Smad3 pS204-pS208 peptide^16^ (Nedd4L WW3 – Smad3 complex displayed in Supplementary Figure 3).

## Discussion

During cell-cycle progression, CPEB1 exerts a dual function. Prior stimulation with progesterone, the CPEB1 protein recruits several cofactors to maintain the mRNA in a translationally repressed state. Upon stimulation by progesterone, CPEB1 becomes phosphorylated on S174, resulting in the dissociation of the repression complex and the recruitment of the polyadenylation complex^20^,^21^. At this early stage, a class of mRNAs, such as those encoding Mos, are activated^3^. However, late translational events, like the translation of cyclin B1, require the previous synthesis of Mos and the activation of Cdc2 kinase. This signaling cascade results in the hyper-phosphorylation of CPEB1 and its subsequent labeling for degradation, which is critical for mitotic cell-cycle progression^9^. The mechanism of CPEB1 degradation is conserved, and the peptidyl-prolyl *cis/trans* isomerase Pin1 has been identified as a crucial factor regulating this process in *Xenopus* and in mammalian cells^22^,^23^,^24^. It is remarkable that, prior to CPEB1 phosphorylation, Pin1 already interacts with this protein, with contacts between the WW domain of Pin1 and at least two regions of the CPEB1 N-terminus, named NF1 and NF2. Phosphorylation independent interactions have also been observed for several other Pin1 targets^25^,^26^,^27^. These regions do not contain canonical motifs for WW domain binding, thereby suggesting that the basal interaction of Pin1 with CPEB1 is achieved through weak interactions with enough affinity to localize the Prolyl isomerase in the scenario. This hypothesis is supported by the identification of the Pin1 residues affected by the proximity of the NF1 and NF2 regions and by the affinity values obtained, which lie in the high μM range. Interestingly, our NMR data revealed that the residues of the Pin1 WW domain participating in the interaction are similar to those involved in the high affinity interaction with phosphorylated motifs. Neither NF1 nor NF2 contained elements of secondary/tertiary structure under our experimental conditions. These constructs consist of 26% and 29% hydrophobic residues, which probably explains the tendency to aggregation displayed by these fragments in solution. Bioinformatic analysis of the sequences predicted the presence of short α-helices, suggesting that these hydrophobic amino acids could interact with the hydrophobic surface of the WW domain (Ile 28 and Trp34), as detected using NMR experiments. The presence of charged amino acids (Asp, Arg and Lys residues) in the NF1 and NF2 sites could be responsible for the chemical shift perturbations detected in the Glu, Gln and Asn residues of the WW domain upon binding of the NF1 and NF2 fragments. Interestingly, in a previous study on the Pin1-PKC interaction, it was demonstrated that PKC recognition of Pin1 is based on a hydrophobic motif in the C-terminal segment of the substrate and does not require phosphorylation. However, the interaction gains affinity when the Pin1 target sites are phosphorylated^26^. The basal CPEB1-Pin1 association could ensure spatial proximity of Pin1 prior to CPEB1 hyper-phosphorylation, allowing for a rapid CPEB1 degradation essential for proper cell-cycle progression. Consistent with this hypothesis is the observation that the N-terminal regions NF1 and NF2, shown to be involved in Pin1 binding, comprise the phosphorylatable binding sites for Pin1. Upon progesterone stimulation, Cdc2 phosphorylates CPEB1 in six pSerPro motifs. Figure 5e shows a schematic overview of the scenario. NMR titration experiments and ITC assays show that Pin1 WW can recognize the six motifs *in vitro*, with the highest affinities towards pS184 and pS210 sites. It is noteworthy that the preferential Pin1 binding sites, pS210 and pS184, are located very close to the TSG-motif (190–195) that when phosphorylated becomes the SCF^β−TrCP^ E3 ubiquitin ligase binding site^4^. The proximity of pS210 to the TSG-motif may explain its essential role for degradation^4^. Residues in the TSG (G192) motif and close to the pS210 binding site (P214) are mutated in esophagus and pancreatic tumors (Catalogue of somatic mutations in cancer: cancer.sanger.ac.uk)^28^,^29^. Our results reveal that CPEB1 pS210 features a unique characteristic compared to the rest of the potential substrates of CPEB1. It is the only substrate of those tested which, in addition to binding to the WW domain, also interacts with the catalytic site of the PPIase domain of Pin1. These interactions might resemble the *in vivo* interaction of full length Pin1 in which a target site can be recognized by the WW domain, while the catalytic site is bound to a second site for isomerization. Sequence comparison between CPEB1 of many vertebrate species reveals a preeminent conservation of the pS210 and pS184 residues and also of adjacent residues (Supplementary Fig. 1). Furthermore, our ^15^N NMR relaxation data of Pin1 indicates an increased inter-domain interaction when in complex with pS210. The functional implications of this modulation regarding inter-domain flexibility remain to be examined in detail. However, studies by *Namanja et al.*^30^ suggested that the inter-domain interaction provides an intra-protein signaling mechanism, through which the WW domain may tune the binding affinity of the catalytic binding site. It has been previously proposed that conduit stiffening of the linker upon substrate binding by the WW domain serves to regulate the conformations sampled by the catalytic site, allowing a fine-tuning of its remote functional site. Given that CPEB1 N-terminus does not contain well-structured domains, perhaps the role of Pin1 activity—as it has been previously suggested^14^— is to allow CPEB1 to explore additional conformations, some of them optimum for the CPEB1 - SCF^β−TrCP^ interaction and the CPEB1 ubiquitin ligase-mediated degradation.

## Methods

### Cloning of Pin1 and CPEB1 constructs

The Pin1 WW domain (Uniprot: Q13526, Residues: 1–41) was cloned via synthetic (template-free) PCR using partially overlapping, complementary oligonucleotides. This approach was used to remove some rare codons present in the template. The DNA insert was cloned into the pETM-30 expression vector containing an additional Glutathion-S-Transferase (GST)-tag between the His6-tag and the TEV cleavage site. The full-length Pin1 clone was obtained from Addgene (Cambridge, USA, provided by Dustin Maly’s Lab) in ampicillin-resistant pMCSG7 vector containing a His6-tag and the TEV cleavage site. Two fragments from the N-terminal region of CPEB1 Isoform 1 (Uniprot Q9BZB8) NF1 (88–183) and NF2 (196–293) were cloned from cDNA into pETM-11 vectors. All clones were confirmed by DNA sequencing. *Escherichia coli* DH5α strain was used for cloning.

### Protein Expression and Purification

Full-length Pin1 and the isolated Pin1 WW domain were expressed and purified mainly as described by *Ranganathan et al.*^31^ and by *Aragón et al.*^16^ respectively. The NF1 and NF2 fragments were expressed at 20 °C in *E. coli* BL21 (DE3) following induction at an optical density of 0.6 (600 nm) with 0.25 mM IPTG overnight. Cells were lysed using an EmulsiFlex-C5 (Avestin) in the presence of lysozyme and DNAseI at 4 °C. Most of the overexpressed protein was in inclusion bodies and therefore purified under denaturing conditions (6 M guanidium chloride). Purified proteins were dialyzed against a buffer containing 100 mM Tris pH 7.0, 400 mM L-Arg, 20% glycerol, and 2 mM DTT. The samples were digested with TEV protease at room temperature overnight, and further purified by size-exclusion chromatography on a HiLoadTM Superdex 75 16/60 prepgrade column (GE Healthcare). To avoid aggregation and precipitation, proteins were concentrated only up to ∼100 μM.

### CPEB1 Phosphopeptide Synthesis and Purification

All CPEB1 phospho-peptides were synthesized manually using Fmoc-based solid-phase peptide synthesis on a ChemMatrix resin with a coupled Rink-Amide linker (0.25 mmol scale). After TFA-based cleavage, peptides were purified by RP-HPLC chromatography using a SunFire C18 Sephasil preparative column (Waters) using an ÄKTApurifier10 (GE Healthcare Life Sciences). A 10%–30% acetonitrile gradient with isotropic 0.1% formic acid was applied, and peptides had an elution time between 20–25 minutes. Fractions containing the pure peptides were lyophilized and re-dissolved in water, and pH was adjusted to match the protein buffer conditions. The final product was analyzed by MALDI-TOF and assigned using homonuclear 2D-NMR spectroscopy. The assignment of the spectra revealed that these peptides populate the trans configuration in solution, (>95%) even in the absence of the WW domain.

### Isothermal Titration Calorimetry

ITC measurements were performed using a nano ITC calorimeter (TA Instruments) at 12 °C or 25 °C. Protein samples were prepared in 20 mM Tris-HCl pH 7.0 and 130 mM NaCl. All samples were degassed and centrifuged prior to the experiments. Protein concentration was measured in a NanoDrop™ 2000 measuring the UV absorption at 280 nm. Peptide concentration was determined by amino acid analysis. Depending on the expected affinity, sigmoidal curves were optimized by injecting 10- to 15-fold concentrated peptide in 16 × 3 μL steps in a cell containing 190 μL of protein at an adjusted concentration of between 150 and 200 μM , stirring at 200 revolutions per minute (rpm). The delay between injections was set to be of 3 minutes. The NanoAnalyze software (TA Instruments) was used to determine the binding isotherms, assuming a single binding site in each molecule (except for the Pin1 full-length interaction with the CPEB1 pS210 peptide, where one Pin1 molecule could interact with two peptides). Baseline controls were acquired with buffer and pure peptide solutions. Measurements were repeated at least twice.

### Ion Mobility - Mass Spectrometry

Travelling wave ion mobility mass spectrometry experiments were performed on a Synapt G1 HDMS mass spectrometer (Waters, Manchester, UK). Samples were placed on a 384-well plate at 15 °C and sprayed using a Triversa NanoMate® (Advion BioSciences) automated Chip-Base nano-electrospray working in positive ion mode. The instrument was calibrated over the 500–8000 Da m/z range using a Cesium Iodide solution. MassLynx 4.1 SCN 704 and Driftscope 2.1 were used for data processing. Samples of unbound full-length Pin1 or of Pin1 in complex with CPEB1 pS210 (final concentrations of 30–50 μM) were prepared in 20–50 mM NH_4_OAc pH 7.2. Prior to analysis, 1D - ^1^H NMR spectra were acquired in order to check the stability of the samples and to compare them with spectra obtained in NMR buffer conditions. Spray voltage was set to 1.75 kV and delivery pressure to 0.5 psi. A reduction of the source pumping speed in the backing region (5.85 mbar) was set for optimal transmission of high mass non-covalent ions. Cone voltage, extraction cone, and source temperature were set at 20 V, 6 V and 20 °C respectively. Ions passed through a quadrupol mass filter to the IM-MS section of the instrument.

### NMR Chemical Shift Perturbation Experiments

Experiments were recorded at 285 and 300 K using a Bruker Avance III 600-MHz spectrometer equipped with a triple resonance gradient probe. Protein samples of the Pin1 WW and full-length Pin1 were equilibrated in either a buffer containing 20 mM Tris-deuterated, 130 mM NaCl or in 50 mM Na_2_SO_4_, 50 mM NaP, 5 mM EDTA and 1 mM DTT, respectively, and supplemented with 10% D_2_O. The final pH was adjusted to 7 for Pin1 WW and to 6.6 for full-length Pin1. Spectra were acquired using 100–200 μM ^15^N-labeled protein samples equilibrated with progressively increasing amounts of the unlabeled peptide until saturation was achieved. Chemical shift perturbation analyses were performed on CcpNmr Analysis^32^ with a 0.15 weighting of ^15^N with respect to ^1^H.

### Relaxation Measurements

Amide relaxation measurements were acquired on a 500-μM ^15^N-labeled full-length Pin1 sample as described^33^. NMR experimental setup details were essentially as reported^34^. Twelve relaxation delay values (21.6, 54, 108, 162, 270, 432, 540, 702, 864, 1080, 1404 and 1728 ms) were measured to determine T1. To determine T2, ten experiments were recorded with the following ^15^N mixing times: 16.74, 33.48, 50.22, 66.96, 100.44, 117.18, 133.92, 167.40, 184.14 and 200.88 ms. All relaxation experiments were acquired as pseudo-3D experiments and subsequently processed as 2D data sets. Peak integration values were fitted to a two-parameter function as follows:


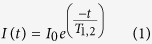


where *I*_*0*_ and *I(t)* are the peak intensities at times 0 and t, respectively.

The rotational correlation time of the Pin1 protein was calculated with equation 2, using the approximation of slow molecular motion τ_c_ larger than 0.5 ns and assuming that only the J(0) and J(ωN) spectral density terms contribute to the overall value. vN is the ^15^N resonance frequency (60,08 × 106 Hz)


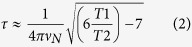


Regarding the ^1^H-^15^N NOE experiment, the reference and the pre-saturated HSQC spectra were acquired in an interleaved manner. The values of the steady-state ^1^H-^15^N NOEs resulted from the ratios of the peak intensities measured in the reference (*I*_*0*_) and the pre-saturated (*I*_*S*_) spectra during the relaxation delay as described^35^. Background noise levels *σ*_*S*_ and *σ*_*0*_ were measured and used to determine the NOE standard deviation through the following relationship:


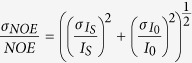


### Structure Determination and Validation

Structure calculation was performed using CNS 1.1^36^. Unambiguously assigned intra- and inter-molecular distance restraints were derived from NOESY experiments (deposited in BMRB; ID: 25569). Backbone dihedral angles φ and ψ restraints were derived from TALOS+^37^. The calculation protocol consisted of an implicit water simulated-annealing of 120 structures using 8,000 cooling steps followed by an explicit water refinement of the calculated structures using all experimental restraints during 1200 steps. The iCing package was used for structure validation. All structure images were generated using PyMOL (http://www.pymol.org/).

## Additional Information

**How to cite this article**: Schelhorn, C. *et al.* Structural Analysis of the Pin1-CPEB1 interaction and its potential role in CPEB1 degradation. *Sci. Rep.* **5**, 14990; doi: 10.1038/srep14990 (2015).

## Supplementary Material

Supplementary Information

## Figures and Tables

**Table 1:** Statistics for the final ensemble of 20 best structures of Pin1 WW—CPEB1pS210 (PDB ID: 2n1o).

**NMR distance and dihedral restraints**	
Inter-molecular	48
Sequential (|i − j| = 1)	54
Medium-range (1 < dist ≤ 4)	34
Long-range (dist > 4)	174
Total	310
Hydrogen bonds	10
Dihedral	73
φ	39
ψ	34
**Structural quality**^a^	
Deviation from idealized covalent geometry	
Bond lengths (Å)	0.01±0.1
Bond angles (°)	0.81±0.1
Improper torsions (°)	2.23±0.1
**CNS potential energy (kcal/mol)**^b^	
Total energy	−1095±45.1
Electrostatic	
Van der Waals	−120.3±10.84
Bonds	−1418±46.11
Angles	125.8±11.29
**Average r.m.s.d. of atomic coordinates (Å)**^c^	
Backbone	0.30±0.06
Heavy atoms	0.96±0.10
**Ramachandran plot analysis (%)**	
Most favorable region	88.0
Additional allowed regions	12.0
Generously allowed regions	0.0
Disallowed regions	0.0

^a^No individual member of the ensemble exhibited distance violations > 0.5 Å or dihedral angle violations > 5°.

^b^For the structural determination we used a full-nonbonded representation during the water refinement, including Lennard–Jones, van der Waals and electrostatic interactions from the OPLSX force field with minor modifications.

^c^Average root-mean-square deviation. of atomic coordinates for residues (15–37) with respect to the mean structure. Pin1 WW regions (9–14, 38–43) were excluded from the statistics because they exhibit a flexible, random-coil conformation.
