# Supplementary material for: Structural Analysis of the Pin1-CPEB1 interaction and its potential role in CPEB1 degradation
Source: Sci Rep. 2015 Oct 12;5:14990. doi: 10.1038/srep14990 (PMC4601027; doi:10.1038/srep14990)
Supplement: Supplementary Information [file srep14990-s1.pdf]

Structural Analysis of the Pin1-CPEB1 interaction and its potential role in CPEB1 degradation

Constanze Schelhorn, Pau Martín-Malpartida, David Suñol and Maria J. Macias

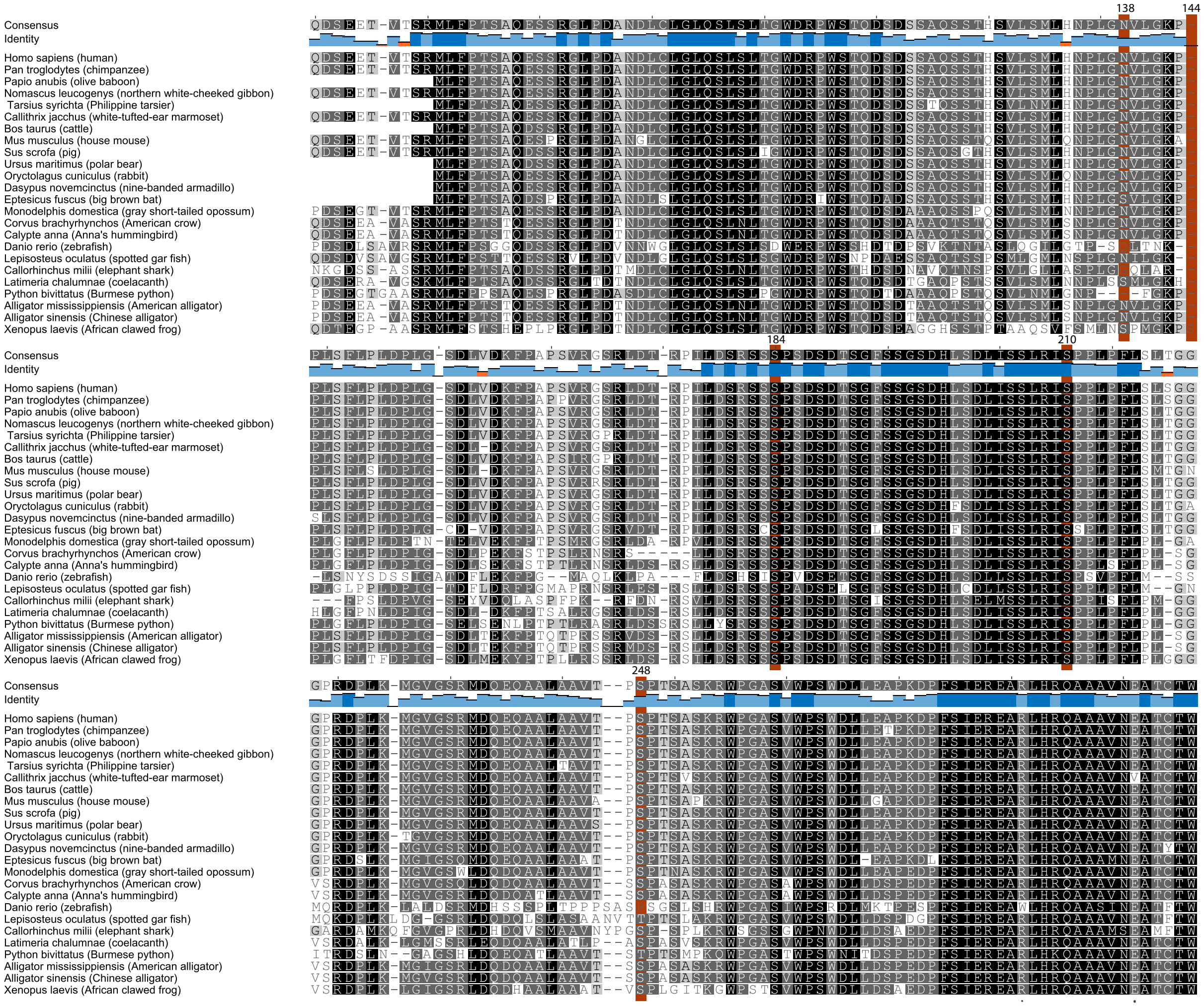

Supplementary Figure 1: Sequence alignment of CPEB1 in vertebrates

Sequence alignment of the N-terminal part of CPEB1. Cdc2 phosphorylation sites within the N-terminal part are indicated by a red box and the sequence numbers referring to the *Xenopus laevis* sequence are indicated. Sequence comparison between CPEB1 sequences of different species reveals a preeminent conservation of the residues corresponding to pS210 and pS184 and adjacent ones.

Peptide	Sequence	MW[Da]
CPEB1 S144	GKPSPLGFLT	1057.2
CPEB1 S248	AATVSPLG	1085.2
CPEB1 $_{p}$S138	SMLN$_{p}$SPMGK	755.81
CPEB1 $_{p}$S144	GKP$_{p}$SPLGFLT	1137.2
CPEB1 $_{p}$S184	SRSS$_{p}$SSDSG	1086.93
CPEB1 $_{p}$S210	LRI$_{p}$SPPLHFLPLGG	1637.9
CPEB1 $_{p}$S210s	LRI$_{p}$SPPLPFG	1175.34
CPEB1 $_{p}$S248	AATV$_{p}$SPLG	835.8
CPEB1 $_{p}$S423	FVR$_{p}$SPSQRLDPG	1393.43

Supplementary Figure 2: CPEB1 peptides
Peptides from CPEB1 N-terminal regions were manually synthesized by Fmoc-Solid Phase Peptide Synthesis. Sequence and molecular weight of all peptides tested are provided.

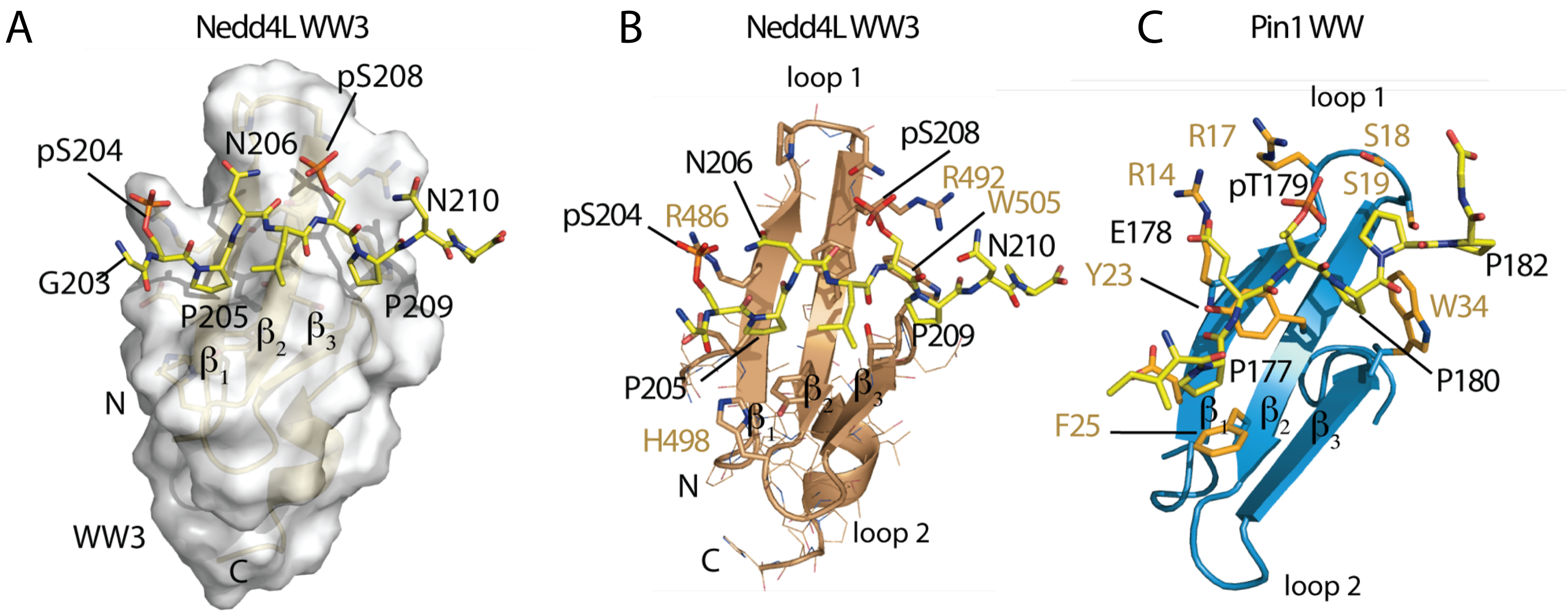

Supplementary Figure 3: Nedd4L WW3 - Smad3 pS204 pS208 NMR solution structure:

A) Nedd4L is shown in semitransparent surface representation, the Smad3 peptide as green sticks

B) Cartoon representation of Nedd4L WW3 domain bound to the di-phosphorylated Smad3 peptide. The orientation of the peptide with respect to the WW domain is resembles the one observed in the complex of Pin1 WW – CPEB1 pS210.

C) Cartoon representation of Pin1 WW domain bound to the Smad3 pT179 [PY] motif. In this complex both Arg^{14} and Arg^{17} are key residues for peptide recognition.
